# 46, XY disorders of sex development combined with aceruloplasminaemia: a case report and review of the literature

**DOI:** 10.1186/s13023-025-03626-2

**Published:** 2025-03-13

**Authors:** Yanju Li, Mei Zhao, Yang Liu, Lan Wang, Yi Huang, Feiqing Wang

**Affiliations:** 1 Department of Hematology, The Affiliated Hospital of Guizhou Medical University, Guiyang, 550001 Guizhou Province China; 2 Clinical Medical Research Center, First Affiliated Hospital of Guizhou University of Traditional Chinese Medicine, Guiyang, 550001 Guizhou Province China; 3 Department of Gastroenterology, The Affiliated Hospital of Guizhou Medical University, Guiyang, 550001 Guizhou Province China

**Keywords:** Disorders of sex development, Aceruloplasminaemia, Whole-exome sequencing, Metabolic abnormality, Iron overload

## Abstract

**Background:**

46, XY disorders of sex development (DSD) and aceruloplasminaemia (ACP) are very rare genetic disorders, and no cases of the coexistence of both disorders have been reported. In ACP patients, iron overload in multiple organs leads to progressive dysfunction of those organs. Early recognition of the coexistence of these conditions is challenging, resulting in difficulties in making a prompt diagnosis and determining the appropriate intervention.

**Results:**

We present a young female patient who was diagnosed with 46, XY DSD due to primary amenorrhea. One decade later, she was admitted for examination due to abnormally high ferritin levels. After the exclusion of common diseases that can cause an increase in ferritin levels, further examination revealed an increase in liver parenchymal density and markedly low CP levels in the plasma. Whole-exome sequencing (WES) revealed a mutation in the CP gene, and the patient was diagnosed with 46, XY DSD with ACP. Iron overload decreased significantly after treatment with deferasirox (DFS).

**Conclusion:**

We aimed to improve the understanding of this complex genetic disorder, and clinicians are advised to be aware of the possibility of coexisting chromosomal abnormalities that emphasize the value of genetic testing, especially in patients with atypical presentations. This information is helpful for identifying other potentially comorbid genetic disorders, achieving the implementation of early treatment strategies, and preventing organ damage.

## Introduction

DSD is a heterogeneous group of congenital conditions that cause a discordance among an individual’s sex chromosomes, gonads, and/or anatomic sex [1]. Some people with DSD possess a 46, XY chromosome complement, collectively referred to as 46, XY DSD [2, 3]. Studies have shown that this chromosomal abnormality can be combined with autosomal chromosome abnormalities/gene mutations [4, 5]. However, DSD combined with ACP has not been reported previously.

Aceruloplasminaemia (ACP) is a rare condition characterized by CP deficiency and iron overload, possibly in the liver, brain, and pancreas. Common manifestations include diabetes mellitus (DM), retinopathy, hepatic disease, and symptoms in the central nervous system (CNS) caused by iron deposition [6, 7].

Here, we report a patient with 46, XY DSD with ACP with the goal of raising awareness of the condition among clinicians who can conduct WES for patients with sex chromosome abnormalities, which can aid in identifying other latent hereditary diseases and implementing early treatment strategies to avoid organ damage.

## Case description, 2014

### Method

*Patient data* A 31-year-old woman presented with primary amenorrhea. Physical examination revealed that she had normal external genitalia, an enlarged clitoris, a patent vaginal orifice and hymen, and a normal vaginal depth of 8 cm.

**Fig. 1:**
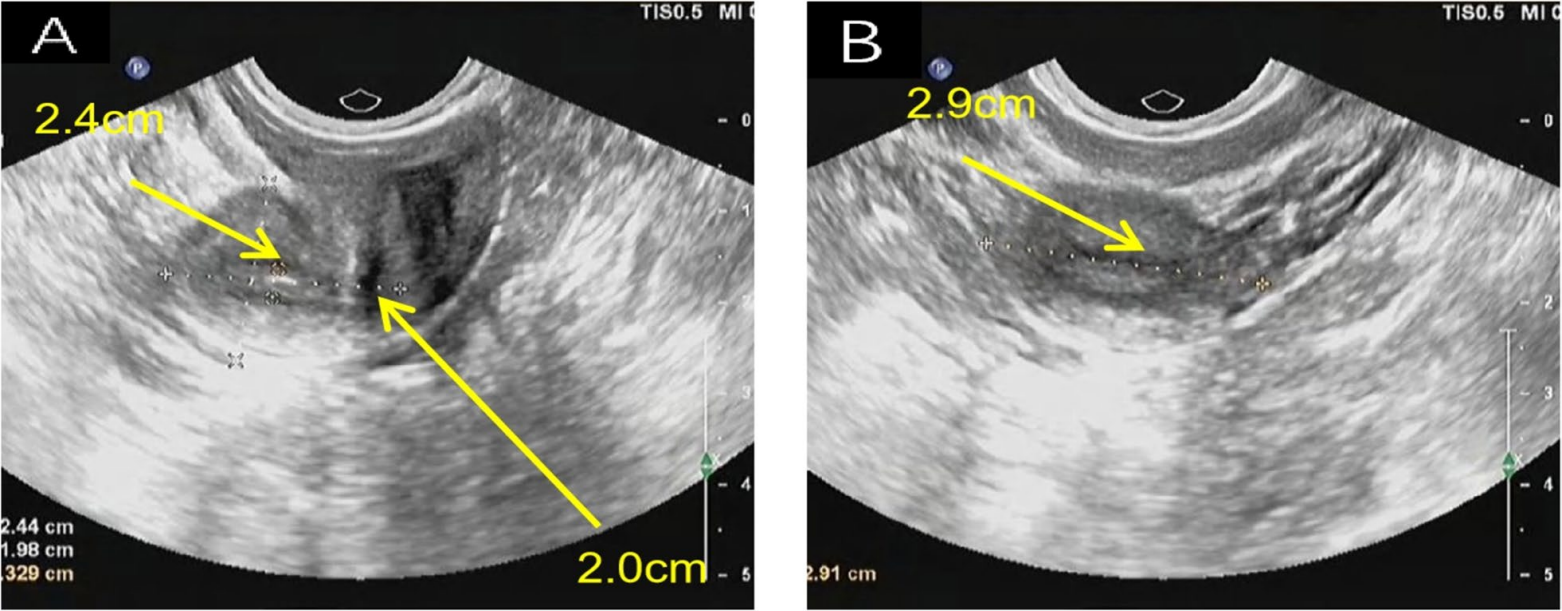
Gynaecological ultrasound images. **A** Longitudinal section of the uterus; **B** transverse section of the uterine body. Gynaecological ultrasound revealed an underdeveloped uterus. The length of the uterine body is approximately 2.4 cm, the diameter of the uterus is approximately 2.0 cm, and the diameter of the uterine body is approximately 2.9 cm. The cervix is relatively long, and the ratio of the length of the uterine body to the cervix is approximately 1:1. The endometrium is approximately 0.33 cm thick. Neither ovary was explored

*Medical imaging and diagnosis* Levels of sex hormones were measured (Table 1). Ultrasound revealed an underdeveloped uterus (anteroposterior position: size = 24 × 29 × 20 mm, endometrial thickness = 3 mm), and the bilateral ovaries were not visible (Fig. 1). Computed tomography of the pelvis revealed a small uterus and no visible testes. Chromosome analyses revealed 46, XY and *SRY* gene positivity. Direct sequencing of the coding region of the *SRY* gene revealed no point mutations. Laparoscopic surgery and bilateral ovarian biopsy were performed. Pathology revealed dysplasia of the left ovary fibrotic hyperplasia and calcification of the right ovary, and the absence of the luteum, corpus albicans, and follicle formation in both ovaries. Therefore, the patient was diagnosed with 46, XY DSD.

**Table 1:** Sex hormone levels

Test	Result	Normal range of value
LH	64.44 IU/L	Follicular phase = 1.90–12.50, ovulation phase = 8.70–76.30, luteal phase = 0.50–16.90
FSH	116.86 IU/L	Follicular phase = 2.50–10.20, ovulation phase = 3.40–33.40, luteal phase = 1.50–9.10
PRL	391.70 mIU/L	Non-pregnant women = 59.00–619.00
E_2_	91.61 pmol/L	Follicular phase = 71.6–529.2, ovulation phase = 234.5–1309.1, luteal phase = 204.8–786.1
PRGE	1.68 nmol/L	Follicular stage = 0.48–4.45, ovulation stage = 10.62–81.28, luteal stage = 14.12–89.14
TSTO	3.14 nmol/L	0.50–2.60

*LH* luteinizing hormone; *FSH* follicle-stimulating hormone; *PRL*: prolactin; *E2* oestradiol; *PRGE* progesterone; *TSTO* testosterone

## Results

*Management* The patient was given cyclic hormone replacement therapy (HRT) with 3 mg/day of oestradiol valerate from Day 1 to Day 21 and 10 mg/day of medroxyprogesterone acetate from Day 11 to Day 21, after which menstruation proceeded regularly.

## Case description, 2024

### Method

*Patient data* In 2024, the patient returned to our hospital due to an abnormally high serum iron level (ferritin > 2000.00 ng/mL [normal range = 13.0–150.0 ng/mL]). The patient did not exhibit neuropsychiatric symptoms, thirst, polydipsia, polyuria, or weight loss. The physical findings were unremarkable. There was no history of consanguineous marriage in the family.

**Fig. 2:**
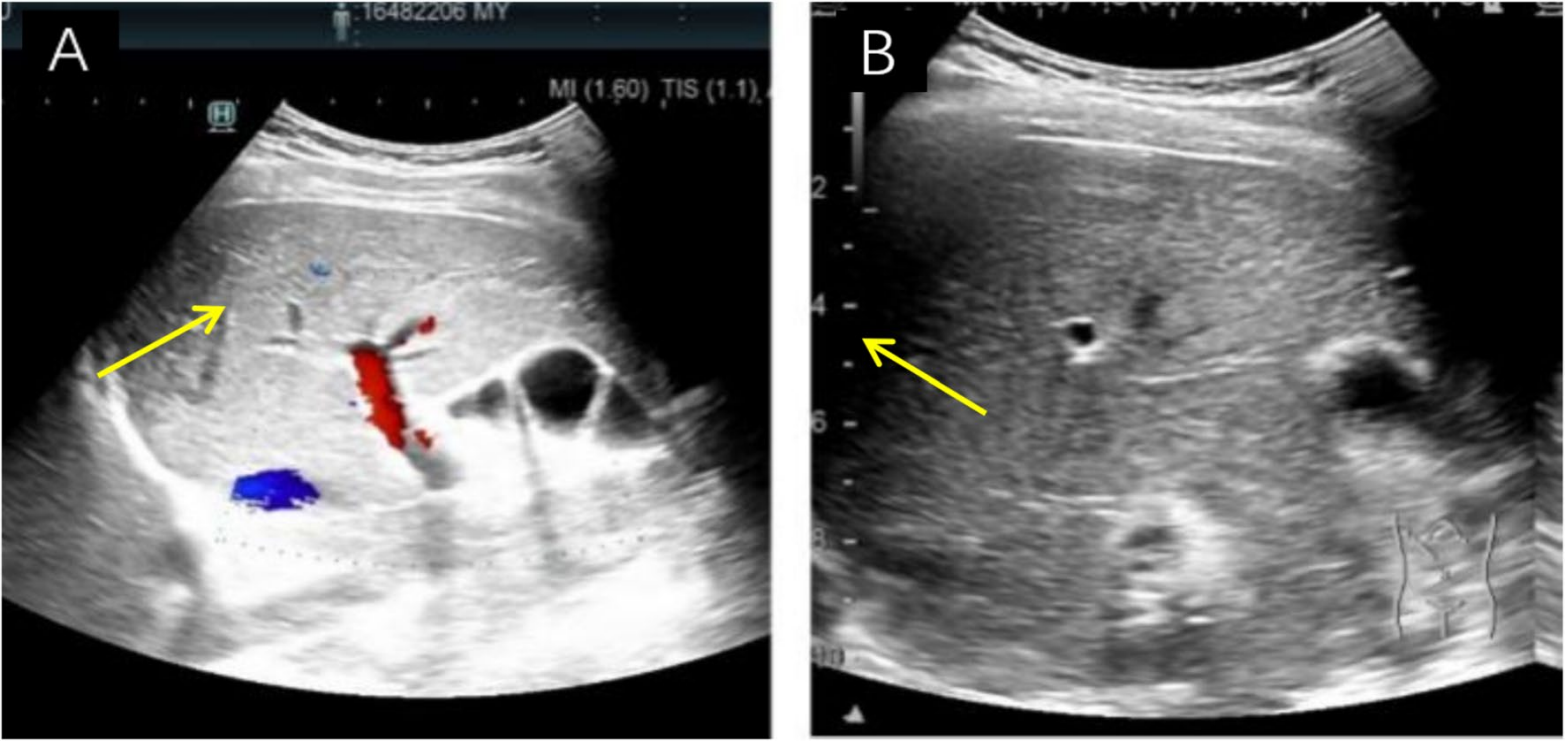
Ultrasound images of the liver. **A** Transection of the right branch of the intercostal portal vein; **B** lower oblique section of the right costal margin. Ultrasound of the liver revealed increased hepatic parenchymal echogenicity. The liver volume is normal

**Fig. 3:**
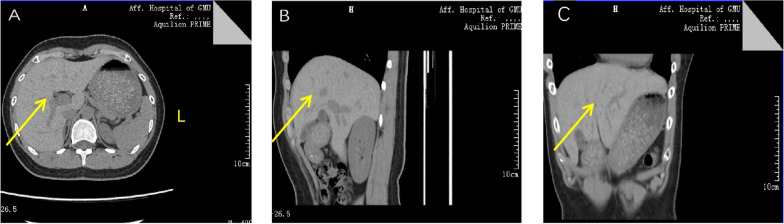
CT images of the upper abdomen. **A** Horizontal position; **B** sagittal position; **C** coronal position. Upper abdomen CT revealed a diffusely increased hepatic parenchyma signal. The liver volume is  slightly enlarged

**Fig. 4:**
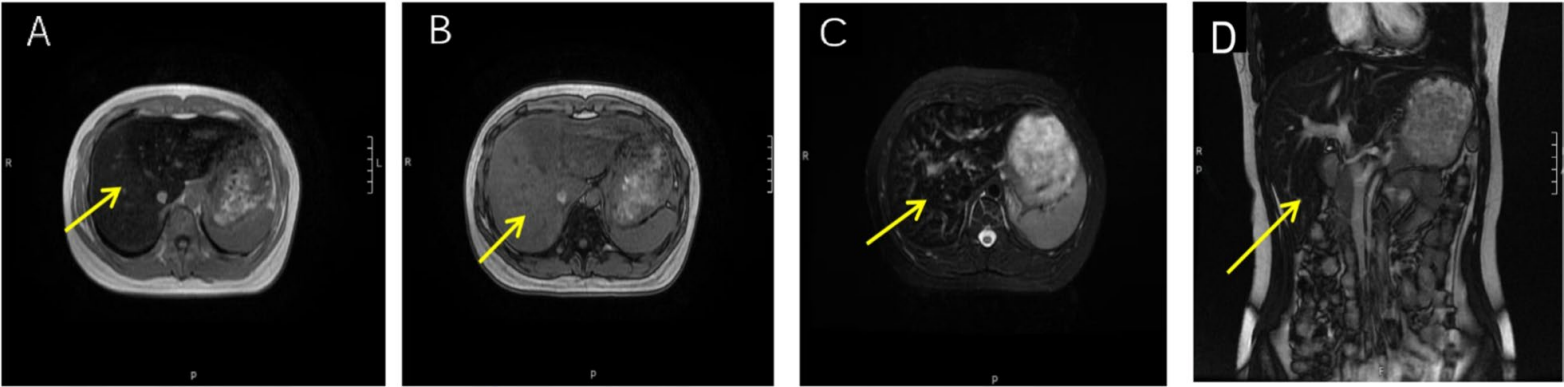
MR images of the upper abdomen in the axial (AX) and coronal (COR) views. **A** T1-weighted (T1W) in-phase images in the AX view showing that the liver signal is diffusely decreased. **B** T1W out-of-phase images in the AX view showing that the liver signal is diffusely increased. **C** T2-weighted (T2W) fat suppression (FS) images in the AX view showing that the liver signal is diffusely decreased. (**D**) T2W images without FS in the COR view showing that the liver signal is diffusely decreased. MR images indicate liver iron overload

*Medical imaging and diagnosis* Serum iron levels, unsaturated iron-binding capacity, total iron-binding capacity, transferrin levels, transferrin saturation, and haemoglobin electrophoresis results were normal. The complete blood count, tumour markers, liver function, renal function, electrolytes, thyroid function, and other common immune-related antibodies were within the normal ranges. Autoimmune liver disease-related antibodies were not detected. Therefore, we were able to exclude common diseases that can cause an increase in the ferritin level. an abdominal ultrasound was subsequently performed; the hepatic parenchymal echo was increased, and no other abnormalities were noted (Fig. 2). Abdominal CT revealed that the liver was slightly enlarged and that the hepatic parenchymal density increased diffusely (Fig. 3). Abdominal magnetic resonance imaging (MRI) revealed that the hepatic parenchymal density was altered diffusely (Fig. 4), so we considered hepatic iron overload in the differential diagnosis. A serum CP level of 4.00 mg/L (normal range = 230–440 mg/L) was recorded. Whole-exome sequencing (WES) revealed a mutation in the *CP* gene: chromosome position, chr3:148,896,290, and variation information, NM - 000096.4:exon16:c.2790C > G (p.Y930); chromosome position chr3:148,925,245 and variation information, NM - 000096.4:exon5:c.941 T > G (p.1314S) (Table 2). This patient was fortunate to exhibit no complications glycated haemoglobin A_1c_ level of 6.1% (normal range = 4.0–6.0%), glycated haemoglobin F, fasting glucose, fasting insulin, C-peptide, 2-h postprandial glucose, insulin, and C-peptide were within normal ranges. The serum levels of type III and type IV collagen, laminin, and hyaluronic acid were normal. The results of T1-weighted MRI and susceptibility-weighted imaging of the brain were normal. Therefore, the final diagnosis was 46, XY DSD combined with ACP.

**Table 2:** Whole-exome sequencing of the CP gene

Gene	Chromosome position	Variation information	MAF	Zygotic state	ACMG rating	Related disease	Genetic pattern
*CP*	Chr3:148 896,290	NM-000096.4:e xon16:c.2790C > G (p.Y930)	–	Heterozygosis	Probable pathogenicity	Aceruloplasminemia	AR
*CP*	Chr3:148 925,245	NM-000096.4:e xon5:c.941 T > G (p.1314S)	–	Heterozygosis	Clinical importance is not known	Aceruloplasminemia	AR

*MAF* mutant-allele fractions; *ACMG* American college of medical genetics and genomics; *AR* autosomal recessive

## Results

*Management* Due to the obvious increase in ferritin levels combined with iron overload in the liver, the patient was administered DFS (500 mg/day p.o.).

*Follow-up* The patient’s ferritin level decreased after 1 month of treatment. She is currently receiving continuous iron chelation therapy. Because her social gender is female, she continues to receive hormone replacement therapy, and we are following the patient’s psychological changes. Youths with gender dysphoria present high rates of anxiety, depression and suicidal ideation, and psychosocial adjustment might be necessary; protective factors that mitigate the risk conferred by gender variance include healthy self-esteem and positive relationships with parents and peers.

## Discussion

46, XY DSD is a rare genetic disease characterized by gonadal dysgenesis and androgen synthesis defects or dysfunctions depending on the underlying mechanism. The genetic background of this disease is complex and involves a variety of genetic factors. Gonadal dysgenesis is related to many genes, such as *SRY, SOX9, MAP3K1* and *NR5A1* or *DMRT1*. Gene mutations may cause abnormal levels of enzymes involved in androgen synthesis [8]. Therefore, the clinical manifestations of 46, XY DSD are heterogeneous.

Single-gene genetic diseases are rare, and the probability of two genetic diseases occurring simultaneously is low, potentially leading to a missed diagnosis or misdiagnosis. The development of genetic testing technology and improvements in clinicians’ understanding of the disease have increased. The occurrence of two rare genetic diseases simultaneously accounts for approximately 4.3% (1.4% ~ 7.2%) of the confirmed cases of genetic diseases [9, 10]. These cases include patients with 46, XY DSD with other congenital anomalies, Frasier syndrome, Denys-Drash syndrome, or congenital diaphragmatic hernia [11–13], but reports of DSD combined with ACP are lacking. Consanguinity and multisystem disease appear to increase the likelihood of multiple genetic diagnoses in a family. Although a definitive causative relationship has not been established, possible genetic or environmental interactions between these two diseases cannot be excluded. Therefore, we conducted a systematic review of ACP research.

*Epidemiology* ACP is an adult-onset autosomal recessive disorder characterized by CP deficiency and iron metabolism disorders, with typical clinical manifestations in the triad of neurological symptoms, diabetes, and retinopathy. Since 1987, more than 130 ACP patients have been reported [14]. According to epidemiologic data derived from estimations in Japan, in which the greatest number of cases has been reported, the incidence of ACP was estimated to be approximately 1/2,000,000 in the offspring of nonconsanguineous marriages [15]. The median age of onset is 40 years, ranging from 25 to 60 years.

*Pathophysiology* ACP is a rare disorder caused by mutations in *CP*. *CP* is located on chromosome 3q24-q25 and contains 20 exons; ~ 70 mutations have been identified in this gene. Most mutations are truncating mutations that cause premature termination of translation [16, 17]. Two isoforms of CP are synthesized via alternative splicing: soluble CP, synthesized by hepatocytes and binding 90% of the copper in the plasma [18], and a glycosylphosphatidylinositol (GPI)-anchored membrane isoform that is involved in crucial iron flux regulation through its ferroxidase function.

In ACP, impairment in the ferroxidase function of CP decreases the efflux of iron stores through the diminished conversion of ferrous iron (Fe^2+^) to ferric iron (Fe^3+^). Ferroxidase activity is necessary because only Fe^3+^ can be incorporated into plasma transferrin and delivered to other cells; therefore, iron bioavailability is decreased in ACP patients [19]. The accumulation of Fe^2+^ can generate dangerous levels of ROS, leading to increased oxidative stress and lipid peroxidation and contributing to the neurodegenerative process in ACP [20].

*Clinical Features* Patients with ACP caused by homozygous mutations exhibit progressive neurodegenerative disease, anaemia, and diabetes and are usually diagnosed late in life upon investigation of anaemia, high ferritin levels, or movement disorders; however, heterozygous mutations are less characterized and believed to be silent [21]. Currently, the neurological phenotype of ACP has been described mainly in Japanese patients. This classical phenotype consists of cerebellar ataxia, hyperkinetic movement disorders and cognitive decline. Caucasian ACP patients present with a wide range of movement disorders [22]. DM was the initial clinical feature of ACP. Studies have shown that Fe overload can increase the risk of diabetes. Fe deposition in the liver leads to oxidative stress disorders, increases apoptosis, decreases the expression of IRS2 and GIUT2 in the liver, causes insulin resistance, and eventually leads to abnormal glucose metabolism [23]. In a systematic review of 55 ACP patients, 68.5% of patients had DM as the first symptom of ACP occurring at a median age from 38.5 to 39.5 years [24]. Although insulin and C-peptide levels were within the normal ranges, the Stumvoll first- and second-phase insulin secretion disposition indices were very low, indicating low beta-cell function in the patient [25].

*Laboratory tests* Laboratory test results indicative of ACP include an extremely low/undetectable serum CP level, an abnormally increased ferritin level, and normal levels of copper in the serum, urine, and liver. Neuroimaging reveals neuron loss and iron deposition in affected areas (especially the basal ganglia). The deposition of iron ions causes signal reduction on T2-weighted MR images, and the degree of reduction is positively correlated with the degree of iron deposition. The transverse relaxation rate (R2) is highly sensitive to changes in iron concentration in the brain and can be used as a noninvasive representation of iron content in the brain. Pathologic examination of the brain tissue of patients with ACP revealed iron deposition in neurons and astrocytes; loss/necrosis of neurons in the iron accumulation area [26]; and neurodegenerative changes in the cerebral cortex, basal ganglia, dentate nucleus, and cerebellum. Postmortem studies have revealed large iron deposits in endocrine sites of the pancreas, accompanied by a significant reduction in pancreatic islet beta cells [27], which also causes insulin-dependent DM. In our patient, liver biopsy revealed that the liver structure was normal with no cirrhosis or fibrosis, but iron deposits were visible in hepatocytes and reticuloendothelial cells.

**Fig. 5:**
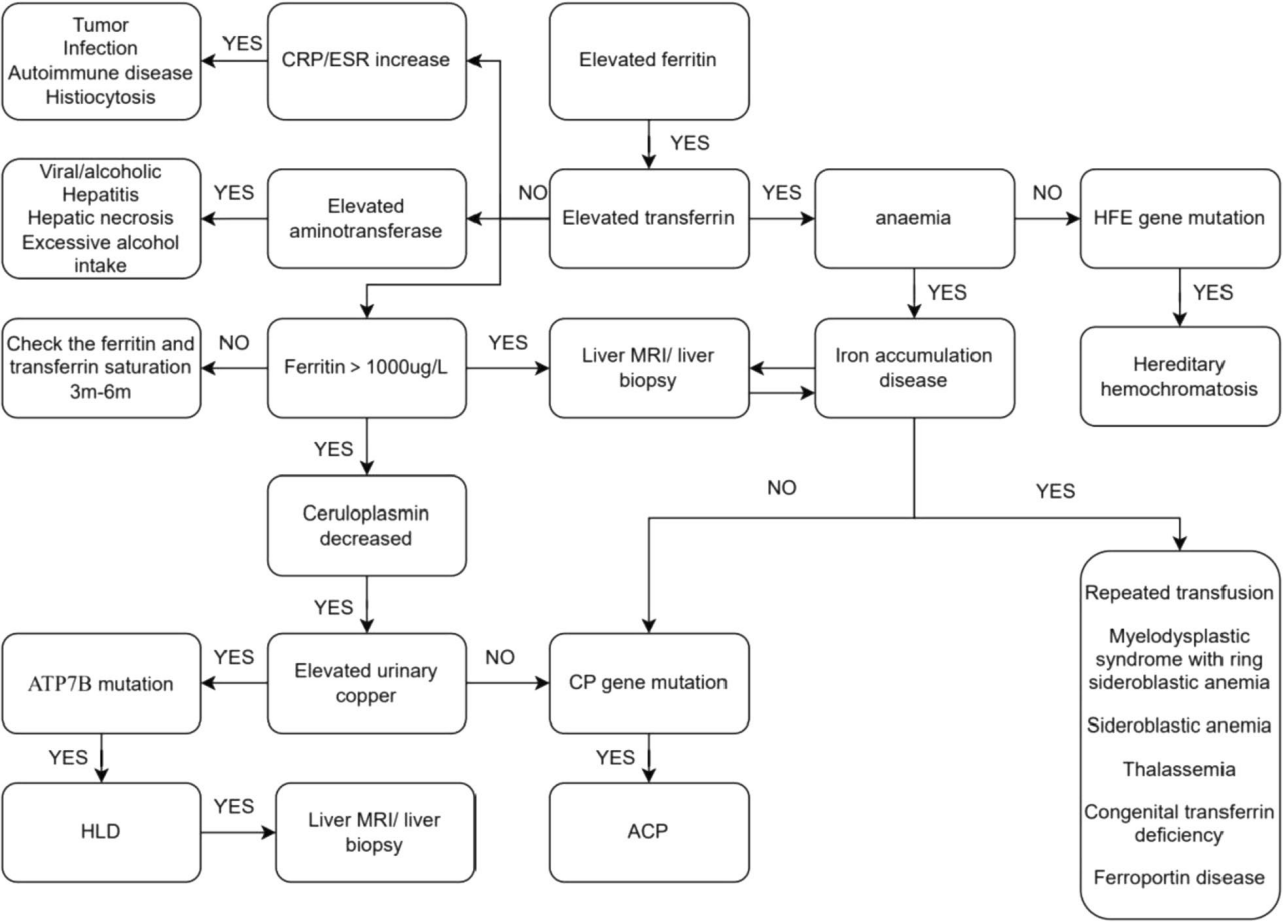
Clinical diagnosis map of elevated ferritin. *CRP* C-reactive protein; *ESR* erythrocyte sedimentation rate; *MRI* magnetic resonance imaging; *CP* ceruloplasmin; *HLD* hepatolenticular degeneration; *ACP* aceruloplasminemia

*Diagnosis* The diagnosis of ACP depends on genetic testing. ACP must be distinguished from other metabolic disorders that cause an abnormal increase in ferritin levels and/or a decrease in CP levels. Our clinical diagnostic process for elevated ferritin levels is shown in Fig. 5.

*Treatment* A specific efficacious treatment for ACP is lacking. Iron chelators are the most common treatment, but some patients have unsatisfactory improvements in neurological symptoms. If a patient has functional damage to various organs (especially CNS damage), treatment methods are not efficacious, and damage is difficult to reverse. Therefore, clinicians should increase their awareness of ACP. Early diagnosis and treatment can aid in disease recovery.

## Conclusion

If ACP and 46, XY DSD can be diagnosed simultaneously, ferritin monitoring can be initiated at an early stage. If necessary, iron chelation therapy can be initiated to effectively prevent injury to related organs. Therefore, we suggest that clinicians should conduct WES for patients with sex chromosome abnormalities (not limited to 46, XY DSD), which can aid in identifying other potentially comorbid genetic disorders and in the early detection, prevention and treatment of specific genetic diseases. WES has positive effects on improving long-term quality of life and prolonging patient survival.

### Limitations

We acknowledge the limitations of this study, including the retrospective design and variable genetic testing practices. In the future, studies with larger sample sizes, standardized genetic testing protocols, functional studies and genotype‒phenotype correlations are needed to refine our understanding of the genetic and clinical spectrum of DSD and ACP.

## Data Availability

The raw data supporting the conclusions of this article will be made available by the authors without undue reservation.

## Abbreviations

DSD: Disorders of sex development
ACP: Aceruloplasminaemia
CP: Ceruloplasmin
WES: Whole-exome sequencing
HRT: Hormone replacement therapy
DFS: Deferasirox
DM: Diabetes mellitus
CNS: Central nervous system
GPI: Glycosylphosphatidylinositol
